# The Role of Nociceptive Neurons in the Pathogenesis of Psoriasis

**DOI:** 10.3389/fimmu.2020.01984

**Published:** 2020-09-29

**Authors:** Xuan Zhang, Yanling He

**Affiliations:** Department of Dermatology, Beijing Chao-Yang Hospital, Capital Medical University, Beijing, China

**Keywords:** psoriasis, nociceptive neurons, neurogenic inflammation, Th17, TRPA1 and TRPV1 receptors

## Abstract

Psoriasis is a chronic inflammatory skin disease. Emerging evidence shows that neurogenic inflammation, induced by nociceptive neurons and T helper 17 cell (Th17) responses, has a fundamental role in maintaining the changes in the immune system due to psoriasis. Nociceptive neurons, specific primary sensory nerves, have a multi-faceted role in detecting noxious stimuli, maintaining homeostasis, and regulating the immunity responses in the skin. Therefore, it is critical to understand the connections and interplay between the nociceptive neurons and the immune system in psoriasis. Here, we review works on the altered innervation that occurs in psoriasis. We examine how these distinct sensory neurons and their signal transducers participate in regulating inflammation. Numerous clinical studies report the dysfunction of nociceptive neurons in psoriasis. We discuss the mechanism behind the inconsistent activation of nociceptive neurons. Moreover, we review how neuropeptides, involved in regulating Th17 responses and the role of nociceptive neurons, regulate immunity in psoriasis. Understanding how nociceptive neurons regulate immune responses enhances our knowledge of the neuroimmunity involved in the pathogenesis of psoriasis and may form the basis for new approaches to treat it.

## Introduction

Skin is a highly sensitive organ that is abundantly innervated by primary sensory nerve endings whose cell bodies are located within the dorsal root ganglia (DRG) and the cranial sensory ganglia (1). The skin contains specific sensory neurons, called nociceptive neurons, that respond to a wide range of noxious stimuli. These stimuli include extreme mechanical stimuli, chemical irritants, and harmful temperature stimuli, which are all capable of causing tissue injury (2). Nociceptive neurons transmit action potentials that induce the pain sensation and trigger the withdrawal reflex (3). Nociceptive neurons also release neuropeptides from peripheral nerve terminals that directly modulate local immune responses (4).

Psoriasis is a chronic inflammatory skin disease characterized by hyperproliferation of the basal epidermal cells, which results in erythematosquamous plaques (5). Clinical reports show spontaneous improvement of psoriatic skin lesions after central or peripheral nerve damage (6), indicating that the nervous system may play an essential role in the development of psoriasis. Moreover, several clinical studies have shown that patients with psoriasis, who suffered insomnia, depression, and anxiety, pointed to these stressors as the main causes of exacerbation of their psoriasis (7–9).

Here we will identify the change in innervation that occurs in patients with psoriasis. We will review the ways nociceptive neurons sense the local environment in skin tissue and then evaluate the dysfunction of peripheral sensory neurons in psoriasis. We will also discuss how the neuropeptides that are released by neurons contribute to the regulation of Th17 immune responses as well as examine the role of nociceptive neurons in a psoriatic mouse model. A proper understanding of the role that nociceptive neurons play in shaping the course of psoriasis could have profound implications for our knowledge of psoriasis pathogenesis and may improve our ability to treat the condition.

## Nociceptive Neurons Sense the Tissue Microenvironment

Similar to the immune system, the peripheral nervous system can detect stimuli directly from the local immune microenvironment. These stimulations can induce excessive excitement and even cause action potentials in nociceptive nerve fibers that evoke pain and/or itching and hyperalgesia. These sensations are produced through various receptors and ion channels, such as transient receptor potential (TRP) channels and inflammatory factor receptors, which are located in free nerve fiber endings.

The TRP channel family, the largest group of noxious stimulus detectors, are Ca^2+^ permeable channels that play a key role in pain sensation (10). In this review, we will focus on and thoroughly investigate two of the TRP channel subfamilies, transient receptor potential cation channel subfamily V, member 1 (TRPV1) and transient receptor potential cation channel subfamily A, member 1 (TRPA1). TRPV1 is a non-selective, ligand-gated, cationic (mainly Ca^2+^) channel responsive to noxious thermal stimuli in the temperature range above 43°C. In contrast to TRPV1, TRPA1 is a non-selective, ligand-gated, Ca^2+^ channel that responds to noxious cold stimuli below 17°C (11). TRPA1 is almost exclusively found in TRPV1-expressing populations of sensory neurons (12). Numerous studies have found that there is cross-sensitization and cross-desensitization between TRPV1 and TRPA1 channels (13–15). Recently, it has been reported that a membrane adapter protein called Tmem100 might form a heteromeric channel construct, playing a fundamental regulatory role in TRPV1-TRPA1 interaction (16).

TRPV1 and TRPA1 sensitization occurs through a mechanism involving multiple protein kinases, such as protein kinases C and A (PKC and PKA, respectively) and Ca^2+^/calmodulin dependent kinase II (CAMKII) (17). Elevated PKC, PKA, or CaMKII activity is associated with sensitization and activation in the nociceptive neurons (18, 19). Numerous studies have reported that inflammation mediators can induce inconsistent activation of nociceptive neurons through G protein-coupled receptors (GPCRs) or receptor tyrosine kinases (RTKs) *via* cAMP/PKA, PLC/PKC, and CaMKII second messenger-signaling pathways (17, 20). These include cytokines [tumor necrosis factor (TNF)], interleukins (IL-1b, IL-6, IL-17A), chemokines, neuropeptides [calcitonin gene-related peptide (CGRP), substance P (SP), and Vasoactive intestinal peptide (VIP)] (21–27). These phenomena provide a treatment target for alleviating skin discomfort and regulating the immune response which will be discussed below.

## Innervation in Psoriasis

The expression of TRP channels (TRPV1, TRPA1) is elevated in psoriatic skin (28, 29). TRP channels are also expressed on non-neuronal cells (30) and, at present, clinical studies targeting the change of nociceptive neurons in psoriasis are still unavailable. Therefore, we have settled for a review of the variation of the primary sensory nerve system.

### Altered Quantity of Nerve Fibers in Psoriasis

Numerous studies have shown that, for every measurable aspect, innervation in psoriatic lesions is higher than in non-psoriatic skin, including the total number of nerve fibers (31), density (31–33), total length (34), and the proportion of nerve fiber penetration into the epidermis (35). However, other investigators have reported conflicting results. It has been reported that protein gene product 9.5 (PGP9.5)-positive nerve endings are completely absent in long-established psoriatic lesions (36). The number of PGP9.5^+^ epidermal immunoreactive nerve fibers was also decreased in highly inflamed psoriatic skin areas (37). These differences may be due to variations in the progression of psoriasis and the duration of time the lesion has been present on the skin.

Nerve ending density is regulated by neurotrophins and other factors of nerve reduction. Nerve growth factor (NGF) and the epidermal growth factor, amphiregulin, facilitates the outgrowth of nerve endings (38, 39), while semaphorin-3A acts as a negative regulator of nerve fiber growth (40). The altered innervation in psoriatic lesions is due to changes in the balance of expression of nerve growth regulators. Related studies have reported that, in psoriatic lesions, the expression of semaphorin-3A is decreased (31, 33, 41) and the expression of amphiregulin (35, 41), NGF (33, 34, 41), tropomyosin receptor kinase A (TrkA) (35, 41), and P75 neurotrophic factor receptors (p75NTR) (42) are enhanced. It was also confirmed, in a psoriasis-like mouse model, that anti-neurotrophins treatment can reduce innervation in and significantly relieve psoriasis-like lesions, suggesting that neurotrophins participate in the pathogenesis of psoriasis by regulating the growth of nerve fibers (43).

### Neuropeptide Content in Psoriasis

In the skin, neuropeptides are mainly synthesized and secreted by C fibers, however, a small part of Aδ fibers and autonomic nerve fibers can express neuropeptides (44, 45). Neuropeptides can also be released by keratinocytes, microvascular endothelial cells, Merkel cells, fibroblasts, and leukocytes under certain physiological conditions (46). Several immunohistochemical studies have demonstrated a change in the expression of neuropeptides in psoriasis. It has been reported that the number of peptide-containing nerve fibers in psoriatic epidermal tissue is elevated as compared to non-psoriatic nerve tissue. The number of SP^+^ nerve fibers increased by six, and the number of CGRP^+^ nerve fibers doubled (33, 47). Increased NGF is responsible for these elevations (48). It has been confirmed that the content of CGRP and SP in psoriatic lesions in psoriasis is elevated (35), accompanied by an increased expression of their receptors (41, 49). Moreover, the content of neuropeptides in the plasma of patients with psoriasis is also elevated, which corresponds with the psoriasis area and severity index (PASI) scores (50, 51).

However, there are conflicting reports that the quantity of peptidergic nerve fibers are significantly decreased in psoriatic lesions (37). It was recently reported that in imiquimod (IMQ)-induced psoriasis-like lesions, there was an increase in the density of non-peptidergic nerve fibers while the density of peptidergic nerve fibers decreased (43). We can speculate that the IMQ-induced psoriasis-like mouse model is similar to the advanced stage of psoriasis, while the decrease in peptidergic nerve fibers is caused by neuropeptides release.

### Abnormal Nociceptive Neurons Function in Psoriasis

Nociceptive neurons play a role in generating pain and pruritus sensations. Abnormal function of these neurons can cause unpleasant skin symptoms in patients. It was found that nearly 90% of patients with psoriasis suffered skin symptoms, including pruritus, discomfort, and hyperalgesia (52). Pruritus is the most common symptom of psoriasis with about 64 to 84% of patients complaining of itching (28). Nearly half (43.6%) of patients experience pain in the lesion area, accompanied by a decreased pressure pain threshold, especially in the scalp and palm areas (53). Similarly, they had lower cold pain thresholds (54). Furthermore, patients with psoriasis who regularly experienced a decreased cold sensation threshold but an increased thermal threshold also had an increased sensitivity to temperature change (55). Moreover, there was a tendency for the pressure and pain thresholds to be decreased in the non-lesion areas of patients with psoriasis (53, 54). The results of these studies show that, in psoriasis, nociceptive neurons fail to consistently transmit sensory signals in lesion as well as non-lesion areas.

Mild systemic inflammation may contribute to the abnormal nociceptor function that is observed in psoriasis (56). The improved PASI scores as well as an improvement in pain intensity are observed after systemic therapy (57), which indicates that abnormalities in sensory function are related to the course of the condition rather than to organismic malformations of the nervous fibers. It is mentioned above that the increased inflammatory factors observed in psoriasis can induce abnormalities in the sensitivity of nociceptive neurons. Research has confirmed that, compared to healthy controls and non-itchy or non-pain lesions, pruritic psoriatic skin contains elevated gene transcription levels of IL-17, IL-23, and IL-31 (28) and hyperalgesia psoriatic skin has higher expression levels of IL-33 (53). IL-17, IL-31, and IL-33 are all important inflammatory factors in the pathogenesis of psoriasis (58). Animal experiments have confirmed that IL-33 can induce dysfunction in sensory nerves (59). Furthermore, increased IL-33 can trigger endothelin-1 (ET-1)-induced secretion of prostaglandin E2 (PGE2) and activate TRP channels in psoriatic lesions, resulting in hyperalgesia (60). Recently, biological treatments targeting psoriatic pro-inflammatory factors have been shown to significantly alleviate symptoms of skin discomfort. For example, treatment with secukinumab and ixekizumab, a monoclonal antibody that selectively neutralizes IL-17A, resulted in a reduction of psoriasis-related scalp pain and itching (61, 62). Additionally, apremilast, an oral small molecule phosphodiesterase-4 inhibitor that elevates cAMP levels in immune cells, downregulates the production of pro-inflammatory mediators, such as TNF-α, IL-17, and IL-23 and increases the production of anti-inflammatory mediators (63). Apremilast also provides rapid and sustained improvement of pruritus and discomfort and pain of the skin (64). These studies support the speculation that the abnormal nociceptive neuronal function found in psoriasis maybe a result of increased pro-inflammatory factors.

Excessive innervation in psoriatic lesions is considered another important cause of itching and hyperalgesia. It was reported that there is a clinical correlation between skin sensitivity and increased nerve fiber density (65). The number of PGP9.5^+^ nerve fibers in psoriatic lesions is positively correlated with pruritus intensity (VAS score) (66). Furthermore, animal experiments show that decreased nerve innervation is accompanied by pruritus alleviation in mice (43). Elevated NGF levels, also contribute to pruritus and hyperalgesia. It was reported that itching in psoriatic lesions is accompanied by an elevation in NGF levels (67). Moreover, local injection of NGF in human skin can directly lead to a reduction in both thermal/cold pain thresholds and mechanical/electrical pain thresholds (68). Apart from prompting the growth of nerve fibers, NGF also can directly induce increased axonal excitability of sensory nerve fibers leading to hyperalgesia (69).

## Nociceptive Neurons Regulate Immune Response in Psoriasis

TRP channels are often co-expressed with the neuropeptides CGRP, SP, and VIP. The TRPA1/TRPV1-evoked release of neuropeptides can cause neurogenic inflammation (11, 70). Here we will focus on the regulation of neuropeptides on Th17 immune responses, which play an important role in the inflammatory pathology observed in psoriasis (71, 72).

### Neuropeptides Prompt Th17 Immune Responses

Functional neuropeptide receptors, such as NK1R (73), RAMP (74), vasoactive intestinal polypeptide receptor 1 (VPAC1), and VPAC2 (75) are expressed on T cells.

In the presence of TGF-β, SP can prompt Th17 cell differentiation through mediation by NK1R (76, 77). Moreover, SP and HK-1, mediated through NK1R, can prompt Th17 to produce IL-17 (78). NK1R expression on Th17 cells can in turn be upregulated by IL-17A, indicating a positive loop between IL-17A and SP (79). It has recently been confirmed that NK1R signaling is necessary to sustain Th17 cell survival and to maintain efficient immune function (73). CGRP has the same function. By binding to RAMP1 on Th17, CGRP directly upregulates IL-17 production and the expression of IL-23R (80). It has been confirmed *in vivo* that, IL-17 production is significantly suppressed in T cells from T cell-specific RAMP1-deficient mice (80).

VIP also plays a role in Th17 differentiation and function through its two receptors, VPAC1 and VPAC2. However, the role of VIP is still under debate. *In vitro*, VIP-VPAC1 axis signals can bias the CD4 T cell response toward a Th17-rich inflammatory type response, in the presence of TGF-β (81). Furthermore, during the onset of Th17 differentiation, VIP prompts the upregulation of the STAT3 gene interaction with the VPAC1 receptor. Moreover, through the VPAC1 and VPAC2 receptors, VIP modulates the upregulation of the transcription factors RAR-related orphan receptor C (RORC), RAR-related orphan receptor A (RORA), and IL-17A genes (75). However, several animal experiments have resulted in conflicting conclusions. In a collagen-induced arthritis (CIA) mouse model (82), a non-obese diabetic (NOD) mouse model (83), and an experimental autoimmune encephalomyelitis (EAE) mouse model (84), VIP suppressed the Th17/Th1-type response. There exists an interesting phenomenon that, based on the same EAE mouse model, two studies selectively knockout (KO) VPAC1 or VPAC2, with conflicting conclusions. In the mice that lacked the VPAC2 receptor, there was an exacerbation of EAE-type clinical, histopathological, and immunological symptoms as well as increased inflammatory Th1/Th17 responses (85). Mice that lacked the VPAC1 gene exhibited a resistance to the development of EAE through the prevention of CNS chemokine upregulation and inflammatory cells infiltration (86). These studies showed that VIP-VPAC1 can enhance Th17 differentiation *in vitro*, while VIP-VPAC2 signals may suppress Th17 responses. Furthermore, the complicated immune environment may interfere with the role of the VIP-VPAC signal in Th17 responses.

Differentiation of Th17 is controlled by T cell receptor (TCR) activation/co-simulation and a distinct set of cytokines, IL-6 and IL-23, participate in terminal differentiation of Th17 cells to help them attain full functionality (87). Numerous studies show that antigen-presenting cells (APC), including monocytes, dendritic cells (DCs), Langerhans cells (LCs), and endothelial cells, express functional neuropeptide receptors, such as RAMP, NKRs, VPAC1, VPAC2, making it possible for neuropeptides to bias APC-induced immune responses (88–90). Tachykinins (SP and HK-1) can enhance the generation of Th17 cells by elevating expression of IL-1b, IL-6, and IL-23 on monocytes (79, 91). It has been confirmed *in vivo* that, SP-NK1R signals can augment the acquisition of MHC II on bone marrow-derived dendritic cells, which then affect Th17 cell infiltration and activity (92). VIP and pituitary adenylate cyclase activating polypeptides (PACAP) have the same effect. *In vitro* exposure of LCs to PACAP and VIP have been found to bias LC antigen presentation toward Th17 cell responses. Furthermore, it was found that, after the application of a contact sensitizer, intradermally administered VIP or PACAP are able to enhance the production of IL-17A from drained lymph node CD4^+^ T cells (93). CGRP is also a Th17 response modifier by way of its actions on APC. CGRP stimulates endothelial cells to produce IL-6, which skews the outcome of the presentation of antigens by LC toward a Th17 response (94). These studies show that the neuropeptides, CGRP, SP, VIP, and PACAP, play an important role in regulating Th17 responses through corresponding receptors that are expressed on APC and Th17 cells.

It has recently been highlighted that the interaction between nociceptive neurons and Th17 responses is involved in psoriasis-like inflammation and Candida albicans (C. albicans) infections. After detection of C. albicans, TRPV1^+^ nociceptive neurons drive IL-23 production by releasing CGRP from dermal CD301b^+^ DCs. CGRP then evokes dermal γδT cells to produce IL-17, which results in protection from C. albicans (95). In a recent study using a transgenic experimental mouse model, it was found that peripheral TRPV1^+^ neuron activation, by isolated stimulation, is able to trigger psoriasis-like dermatitis and type 17 inflammation by local TCR γδ T cells, which can be blocked by botulinum neurotoxin A (BoNT/A) (96). Moreover, onabotulinumtoxinA can significantly decrease PASI and physician global assessment (PGA) scores in patients with psoriasis (97). Both BoNT/A and onabotulinumtoxinA are botulinum toxins (Botox) that can block neuronal vesicle release (98), demonstrating the efficacy of nerve-targeting treatments in psoriasis. This research suggests that TRPV1^+^ nociceptive neurons play a crucial role in the Th17 immune response *via* the release of neuropeptides from free terminals in psoriasis.

### TRPV1^+^/TRPA1^+^ Nociceptive Neurons Regulate Immunity in a Psoriatic Mouse Model

Pharmacological ablation of TRPV1^+^ fibers result in significantly alleviated psoriasis-like lesions accompanied by decreased expression of IL-23, IL-17, and IL-22 and decreased recruitment of inflammatory cells in the murine model of psoriasiform skin inflammation (99). It was further confirmed that key inflammatory factors in psoriatic and epidermal hyperplasia are significantly reduced in TRPV1 KO mice (100).

It is mentioned above that there is cross-regulation of function between TRPV1 and TRPA1. In recent years, the role of TRPA1 in psoriasis has been explored. Kemény et al. reported that TRPA1 KO or TRPA1 antagonists (A967079) treatment can both significantly enhance psoriasis dermatitis and increase hind paw scratching, suggesting that TRPA1 plays a protective role in psoriasis (101). However, Zhou et al. hold the opposite opinion that, compared with wild-type (WT) mice, IMQ-induced psoriasiform dermatitis and Th17-related cytokine expression is significantly reduced in TRPA1 KO mice. The authors attribute this contradictory result to the fact that Zhou used a different protocol than Kemény. Zhou used an original experimental protocol that required a relatively higher dose of IMQ, while Kemény used the Finn chamber, which is characterized as a “localized model.” It is confirmed that high doses of IMQ induce splenomegaly, increased plasma concentration of inflammatory cytokines, and body weight loss indicating systemic inflammatory reactions in mice, which can be avoid in the Finn chamber application (102). As a result, we can speculate that the immune mediation function of TRPA1^+^ nociceptive neurons may be influenced by the systemic immune environment.

## Conclusion

**Figure 1:**
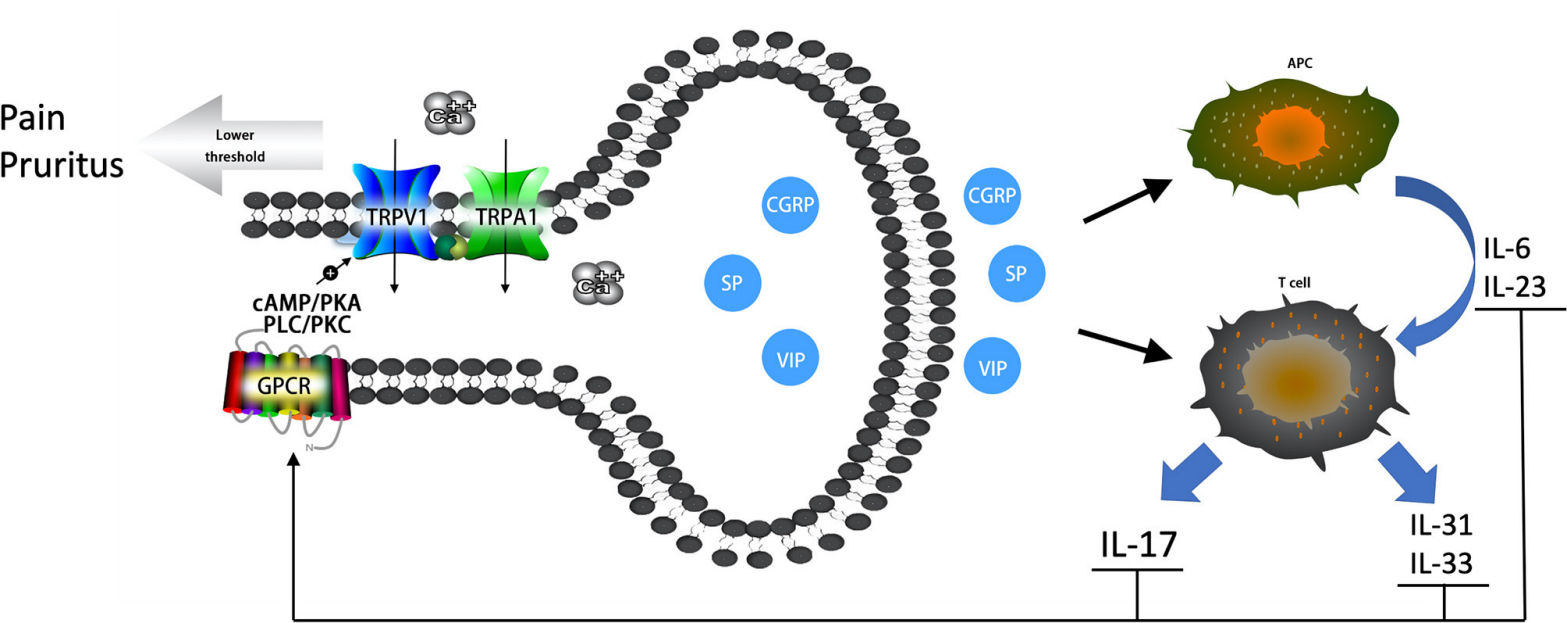
The vicious circular pathway between nociceptive neurons and Th17 immune responses in psoriatic lesions. Neuropeptides (CGRP, SP, VIP) prompt the release of IL-6 and IL-23 and bias antigen presentation for Th17 cell responses. Neuropeptides also prompt Th cells to release IL-17, IL-31, and IL-33. Increased cytokines can sensitize TRPV1 and TRPA1 channels through GPCRs *via* secondary messenger-signaling pathways, the cAMP/PKA and PLC/PKC pathways, following Ca^2+^ elevation. An elevated Ca^2+^ concentrate prompts the release of neuropeptides, which forms a vicious circle pathway between nociceptive neurons and the local immune system. Sensitized TRPV1 and TRPA1 channels result in pruritus, pain, and hyperalgesia experienced by patients with psoriasis.

The primary emphasis of this review has been on examining the role of the nervous system, especially nociceptive neurons, in the pathogenesis of psoriasis (Figure 1). Numerous studies have reported increased innervation of primary sensory nerve fibers and elevated gene expression of TRP channels in psoriasis. However, the dynamic changes of nociceptive neurons in psoriatic lesions are still not fully understood. Moreover, further research is needed to illuminate the role of TRPA1^+^ nociceptive neurons in psoriatic immune responses. The vicious circular pathway between nociceptive neurons and Th17 responses is responsible for pruritus, pain, and hyperalgesia experienced by patients with psoriasis. The effective targeting of this pathway is the reason anti-IL-17 therapy proved most effective in reducing pruritus, while traditional immune system suppressants (methotrexate and retinoids) failed (103). This review also provides a theoretical basis for the formulation of promising nerve-targeting treatments for psoriasis in the future. However, further clinical trials are still needed to understand the effectiveness of Botox. It will be beneficial for future studies to continue to explore the synergy between nociceptive neurons and the immune system in psoriasis, which could result in improved patient outcomes.

## Author Contributions

XZ wrote the manuscript. YH approved the final version of the manuscript. All authors contributed to the article and approved the submitted version.

## Conflict of Interest

The authors declare that the research was conducted in the absence of any commercial or financial relationships that could be construed as a potential conflict of interest.
